# Contribution of the ELFG Test in Algorithms of Non-Invasive Markers towards the Diagnosis of Significant Fibrosis in Chronic Hepatitis C

**DOI:** 10.1371/journal.pone.0059088

**Published:** 2013-03-21

**Authors:** Jean-Pierre Zarski, Nathalie Sturm, Jérôme Guechot, Elie-Serge Zafrani, Michel Vaubourdolle, Sophie Thoret, Jennifer Margier, Sandra David-Tchouda, Jean-Luc Bosson

**Affiliations:** 1 Hepato-Gastroenterology Clinic, DIGIDUNE Pole, Grenoble University Hospital, Grenoble, France; 2 INSERM/UJF U823 IAPC unit, Institut Albert Bonniot, Grenoble, France; 3 Department of Pathological Anatomy and Cytology, Biology Pole, Grenoble University Hospital, Grenoble, France; 4 Biochemistry A Laboratory, Saint Antoine Hospital, Assistance Publique-Hôpitaux de Paris, Paris, France; 5 Pathology Department, Henri Mondor Hospital Center, Assistance Publique-Hôpitaux de Paris, Paris Est Creteil University, Créteil, France; 6 UJF-Grenoble 1/CNRS/Clinical Research Centre-Inserm CIC03/TIMC-IMAG UMR 5525/Themas, Grenoble University Hospital, Grenoble, France; 7 Medical-Economics unit, Clinical Research Administration, Grenoble University Hospital, Grenoble, France; University Hospital of Essen, Germany

## Abstract

**Background and Aims:**

We aimed to determine the best algorithms for the diagnosis of significant fibrosis in chronic hepatitis C (CHC) patients using all available parameters and tests.

**Patients and Methods:**

We used the database from our study of 507 patients with histologically proven CHC in which fibrosis was evaluated by liver biopsy (Metavir) and tests: Fibrometer®, Fibrotest®, Hepascore®, Apri, ELFG, MP3, Forn's, hyaluronic acid, tissue inhibitor of metalloproteinase-1 (TIMP1), MMP1, collagen IV and when possible Fibroscan™. For the first test we used 90% negative predictive value to exclude patients with F≤1, next an induction algorithm was applied giving the best tests with at least 80% positive predictive value for the diagnosis of F≥2. The algorithms were computed using the R Software C4.5 program to select the best tests and cut-offs. The algorithm was automatically induced without premises on the part of the investigators. We also examined the inter-observer variations after independent review of liver biopsies by two pathologists. A medico-economic analysis compared the screening strategies with liver biopsy.

**Results:**

In “intention to diagnose” the best algorithms for F≥2 were Fibrometer ®, Fibrotest®, or Hepascore® in first intention with the ELFG score in second intention for indeterminate cases. The percentage of avoided biopsies varied between 50% (Fibrotest® or Fibrometer®+ELFG) and 51% (Hepascore®+ELFG). In “per-analysis” Fibroscan™+ELFG avoided liver biopsy in 55% of cases. The diagnostic performance of these screening strategies was statistically superior to the usual combinations (Fibrometer® or Fibrotest®+Fibroscan™) and was cost effective. We note that the consensual review of liver biopsies between the two pathologists was mainly in favor of F1 (64–69%).

**Conclusion:**

The ELFG test could replace Fibroscan in most currently used algorithms for the diagnosis of significant fibrosis including for those patients for whom Fibroscan™ is unusable.

## Introduction

Blood tests and transient elastography (Fibroscan™) have been developed with the objective of replacing liver biopsy for the diagnosis of liver fibrosis in chronic hepatitis C (CHC). Retrospective and recent independent prospective studies have shown that the four most validated non-invasive methods, Fibrotest®, Fibrometer®, Hepascore® and Fibroscan™ have similar performances for the diagnosis of significant fibrosis (METAVIR F≥2) in CHC [1]–[5]. These methods have been recently approved after an independent systematic review by the French National Authority for Health for the first line assessment of fibrosis in naïve patients with CHC [6]. Other blood tests have also been proposed for the diagnosis of liver fibrosis in CHC: FIB-4 [7], Forns' score [8], MP3 [9], Apri [10], ELFG [11], and Hyaluronic acid [12]. However, in our recent study their diagnostic performance seemed to be lower than that of the four most validated tests [13].

The performance of these non-invasive methods for the diagnosis of significant fibrosis or cirrhosis may be improved when they are combined, as suggested by recently proposed algorithms. These use either two blood tests sequentially, such as the Sequential Algorithm for Fibrosis Evaluation (SAFE) [14], [15] or are based on agreement between a blood test and Fibroscan™ results, as for the Bordeaux Algorithm (BA) [16]. To date the most used and validated algorithm has been Fibrotest®+Fibroscan™. However, this strategy has some limitations requiring an expensive Fibroscan™ machine that is not always available; it cannot be used in about 10% of cases, often because of obesity, and gives uninterpretable results in another 10% of cases [17]. For this combination the positive predictive value (PPV) and/or negative predictive value (PPV) have not always been determined and number of avoided biopsies was only 30–50% for the diagnosis of significant fibrosis [2], [16]. Moreover, in constructing these algorithms, all the available blood tests had not been introduced in the statistical analysis model. Furthermore the relative cost of the different screening strategies has not been thoroughly analysed.

Using data from the FIBROSTAR study [13] we aim here to determine simple screening strategy algorithms that can be used in routine clinical practice by most physicians with the best accuracy for the diagnosis of significant fibrosis in CHC. We also consider the relative costs of the screening strategies in comparison with liver biopsy in this indication.

## Patients and Methods

### Ethics Statement

The main ‘FIBROSTAR’ study protocol was approved by the regional ethics committee “Comité de Protection des Personnes (CPP) Sud-Est 5” France. All patients gave written informed consent.

### Patients

Our patient population, along with the study inclusion and exclusion criteria, has been previously described [13]. Briefly, treatment naïve consecutive adult patients with histologically proven hepatitis C were prospectively included. Patients with compensated cirrhosis could be included, but those with co-existing liver disease were excluded. Liver biopsies were performed as part of normal clinical care for staging and grading of the liver disease before antiviral treatment.

### Biological Scores of Liver Fibrosis

Blood sampling and handling were previously reported in detail [13] and methods are summarized in Text S1. We emphasize here that cholesterol, platelet count and prothrombin time were immediately measured in each centre; all other biochemical parameters, aspartate aminotransferase (ASAT), alanine aminotransferase (ALAT), gamma glutamyl transpeptidase (GGT), Bilirubin, Urea, Apolipoprotein A1, Alfa-2 macroglobulin, Haptoglobin) were measured in a centralized laboratory. All the tests were performed blind of clinical and histological data.

Each biochemical parameter was firstly evaluated alone then the following blood tests were introduced in the analysis: Fibrotest®, Fibrometer®, Forns score, Apri, MP3, ELFG, Hepascore®, FIB-4, hyaluronic acid and collagen IV [18]. Blood test scores were calculated according to the published formulae, the patent for Fibrotest® or by courtesy of the manufacturer (BioLivescale) for Fibrometer®. The list of variables included in each test and the measurement techniques were previously described [13].

### Liver stiffness measurement by transient elastography (Fibroscan™)

Measurements were made as previously described [13] by the operator who performed the liver biopsy. Liver stiffness measurement (LSM) failure was defined as zero valid shots (after at least 10 attempts) and “unreliable examinations” were defined as fewer than 10 valid shots or an interquartile range (IQR)/LSM greater than 30% or a success rate less than 60% [19].

### Liver biopsy

Liver biopsies and fibrosis scoring according to the METAVIR scale were performed as described by two senior liver pathologists (NS and ESZ) with an inter-observer κ agreement of 0.48 and a weighted κ agreement of 0.75 [13]. Biopsies were examined for steatosis, prevalence of non-alcoholic steatohepatitis and iron deposits. To be considered for scoring, biopsies less than 20 mm had to measure at least 15 mm and/or contain at least 11 portal tracts.

### Statistical Analysis and Automated Algorithm

In first intention we used one of the four tests that have been shown to perform best according to the published studies [13] and that have been validated by the French health authorities (HAS) (Fibrotest® or Fibrometer® or Hepascore® or Fibroscan™) [6] to identify patients with no or mild fibrosis (METAVIR F≤1) using cut-offs given by a 90% negative predictive value (NPV). Then, we constructed C4.5 algorithms using an automated program to determine the most effective second test, with a positive predictive value (PPV) of 80%, to identify patients with significant fibrosis (METAVIR F≥2). For each algorithm we calculated the number of biopsies avoided. The algorithm gave the cut-offs to be used when making clinical decisions and these are consistent with several publications in the field [20].

The C4.5 algorithm was performed on R software (version 2.9.1). It is a decision tree algorithm (statistical classifier) that uses Shannon's entropy measure. At each node, the program chooses the variable that best separates the populations (the difference in entropies must be maximal).The process is then repeated on the subgroups obtained. The algorithm is automatically induced without premises [21].

In a *post hoc* analysis we performed a principal component analysis (PCA) of the main tests: Fibrotest®, Fibrometer®, ELFG, Hepascore®, ELFG and Fibroscan^TM^.

### Medico-economic analysis

To meet current requirements for optimization of health spending, a *post hoc* cost analysis of different screening strategies was conducted. A hospital perspective was chosen and only medical costs were included. As complications related to liver biopsy are heterogeneous and rare (3 per 1,000), they were not included in this analysis. Costs of blood tests were based on reimbursement rates by French Health Insurance (FHI), to which we added the cost of the scoring algorithm where appropriate. For each screening strategy, the described cost included the non-invasive tests plus liver biopsy cost if needed.

Regarding the cost of screening by transient elastography (Fibroscan^TM^) and the cost of the liver biopsy, the cost of reimbursement by FHI was considerably lower than the real cost of performing the procedures by the hospital. Thus we calculated the cost for the hospital then performed a sensitivity analysis. This sensitivity analysis permitted us to vary the costs for liver biopsy and to allow for cost recovery of the medical device (Fibroscan^TM^). Lastly, to take into account the very high variability of the cost of the biopsy and to allow greater transposability of costs from one hospital to another, we set three levels of liver biopsy cost based on published data and the cost in our hospital: 800 Euros, 1,000 Euros and 1,200 Euros. Further details of the economic analysis are provided in Text S2.

## Results

### Patients' characteristics

**Figure 1:**
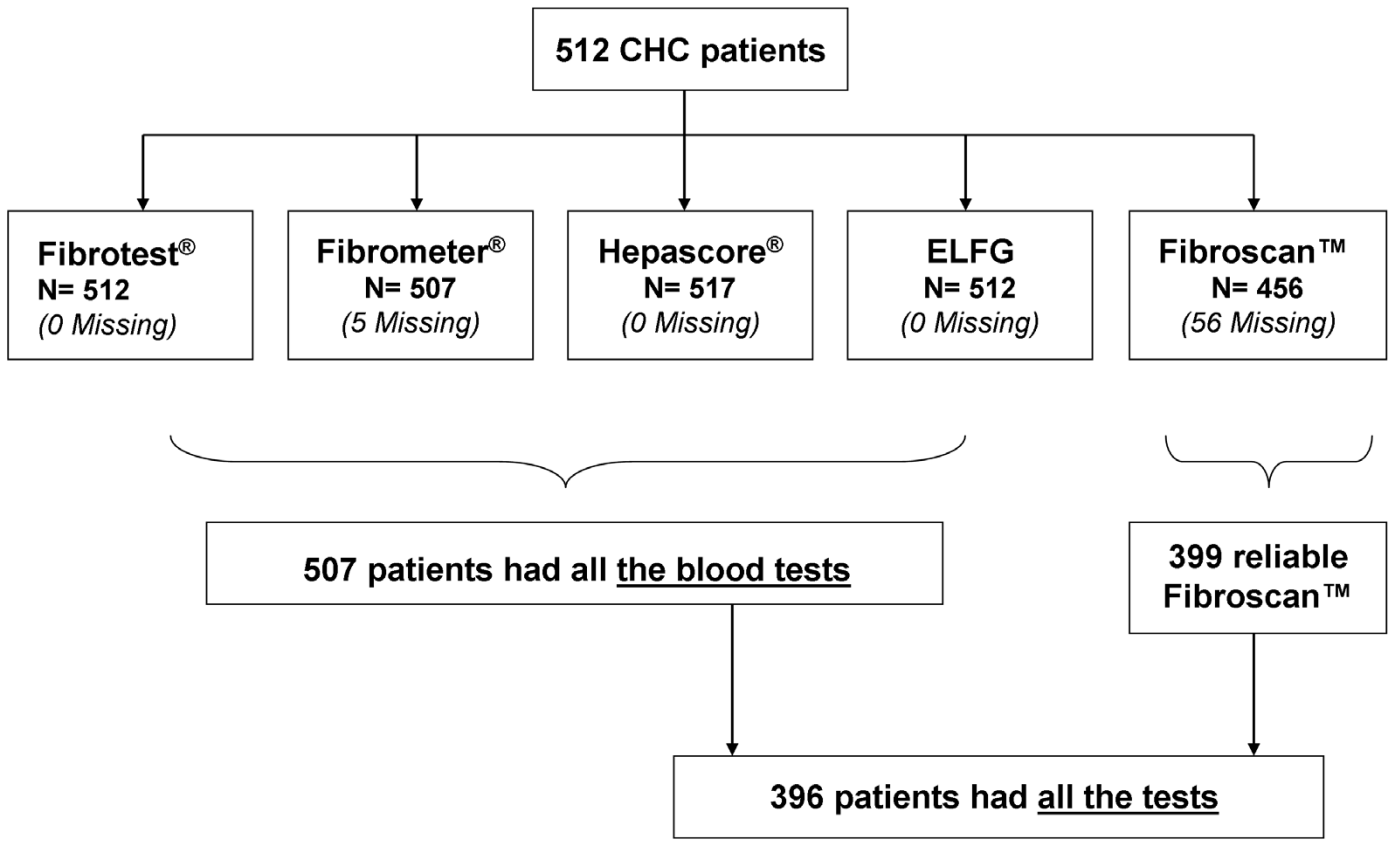
Study Flow Chart. N: number of chronic hepatitis C patients with test results; and the number of patients without the test or with missing test data are shown in parentheses.

Figure 1 shows the flow chart for the 512 patients included in the main study between November 2006 and July 2008. Their main demographic, laboratory and histological features have been previously described (13) and are presented here as Table S1. Table 1 presents the results of different blood tests, selected pertinent parameters and Fibroscan™ in both the intention to diagnose and per-protocol populations. No statistical difference was observed between the two groups regarding these parameters.

**Table 1:** Scores from the different tests and selected parameters for the 507 CHC patients having all the blood tests (intention to diagnose population) and the 396 CHC patients with all the blood tests and reliable Fibroscan^TM^ (per protocol population).

Non-Invasive Test	n = 507	n = 396
Hepascore®	0.5±0.3	0.5±0.3
Fibroscan^TM^	9.7±7.3	9.7±7.3
Fibrotest®	0.5±0.3	0.5±0.3
Fibrometer®	0.6±0.3	0.6±0.3
Apri	0.3±0.4	0.3±0.4
ELFG	−0.8±0.9	−0.8±0.9
MP3	0.3±0.1	0.3±0.1
Hyaluronic acid (µg/L)	69.7±101.5	67.4±99.2
TIMP1* (µg/L)	173.8±69.0	170.5±67.6
MM1**(µg/L)	4.4±3.4	4.2±3.3
PIIINP***(µg/L)	5.4±4.1	5.3±4.4
Collagen-IV (µg/L)	170.5±85.0	170.3±86.9

* TIMP1: tissue inhibitor of metalloproteinase-1; **MM1: matrix metalloproteinase-1;

*** PIIINP: N-terminal peptide of type III procollagen.

### Proposed algorithms

**Figure 2:**
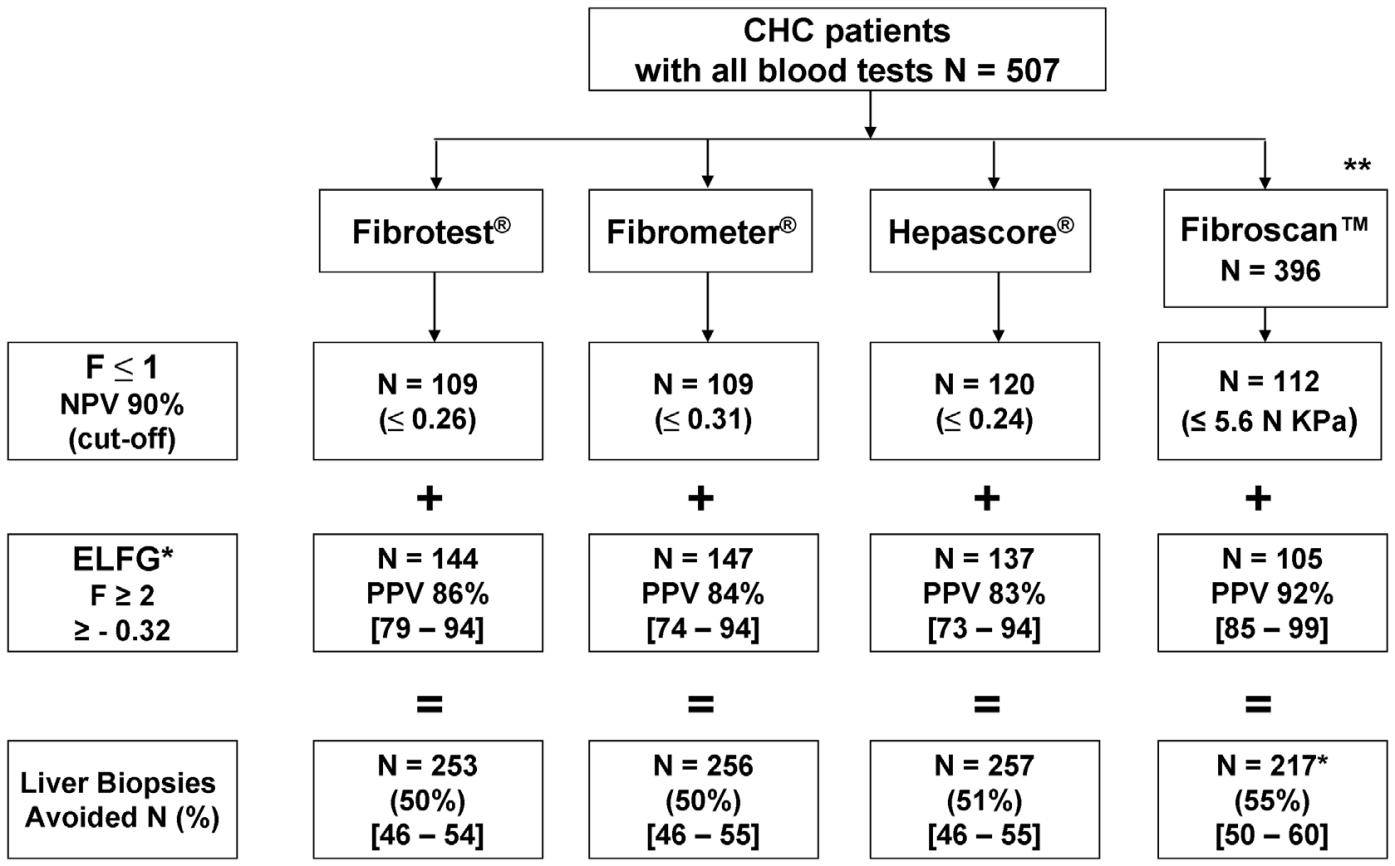
Proposed algorithm: automatically determined by the C4.5 program with the number of avoided liver biopsies. The bottom line gives the total number of liver biopsies avoided following one of the three most validated blood tests or Fibroscan followed by the ELFG test for those patients for whom the first test was not conclusive. N: number of patients; F: Metavir liver biopsy Fibrosis score; NPV: Negative Predictive Value with the cut-off in parentheses; PPV: Positive Predictive Value with the cut-off range in brackets. * = cut-off = >−0.32; ** = per protocol analysis.

The results of different algorithms are presented in Figure 2 with cut-offs for the blood tests and Fibroscan™ and the number of avoided liver biopsies. First we selected and entered the four most validated tests into the model (Fibrotest®, Fibrometer®, Hepascore® and Fibroscan™). The cut-off was determined with a 90% NPV that excluded patients with no or mild fibrosis F≤1. Second, when the value was superior to the cut-off, the computer automatically introduced another test in the model and calculated the PPV, thus giving the number of patients with moderate or severe fibrosis (F≥2). With this method, the ELFG was always chosen by the computer whatever the first test introduced in the model. In the intermediate zone (“impossible to conclude”) we considered that liver biopsy was mandatory. This procedure gave the number of liver biopsies avoided.

We explored the interest of introducing a third test in the model. However we did not observe any significant increase in the number of biopsies avoided when we compared the diagnostic performance of algorithms with 2 or 3 tests (data not shown). Nevertheless, the third test selected by the software was always Fibroscan™.

We also compared the diagnostic performance of our algorithms with the usual combinations published in the literature (Table 2 and Figure S1). The number of avoided liver biopsies was significantly lower with the SAFE algorithm (16%) and higher with the “Bordeaux” algorithm (68%). However the predictive values were lower with this latter combination (NPV: 80% and PPV: 84%)

**Table 2:** Comparison between proposed algorithm, with ELFG, and published algorithms (that include Fibroscan^TM^) in terms of number of patients with avoided liver biopsies.

	Fibrometer®	Fibrotest®	Hepascore®	Fibroscan^TM^
	(N = 507)	(N = 507)	(N = 507)	(N = 396)
ELFG (cut-off:≤−0.32)	256 (50%)	253 (50%)	257 (51%)	217 (55%)
Fibroscan™(cut-off: 5.6 KPa)	109 (21%)	109 (21%)	120 (24%)	–

Finally we considered the inter-observer variations after independent histological analysis of liver biopsies by two pathologists, especially for F1/F2, but also for other lesions considered in the histological examination.

No significant difference was observed for the different algorithms concerning all the histological lesions, especially the number of discordances for F1/F2 staging, between the pathologists (Table 3). Moreover, the quality of the liver biopsy (length, number of portal-tracts, number of septa/length of biopsy), the METAVIR activity index, the rate of steatosis and the presence of steatohepatitis or iron deposits were not statistically different between patients with discordances and those without discordances. The consensual review of liver biopsies by the two pathologists was mainly in favor of F1 (64–69%).

**Table 3:** Characteristics of liver biopsy for each algorithm, when liver biopsy is required, and in the overall population.

	Fibrometer®	Fibrotest®	Hepascore®	Fibroscan™	4 algorithms	Overall
	+ELFG	+ELFG	+ELFG	+ELFG		population
	N = 271	N = 271	N = 259	N = 192	N = 119	N = 507
Length of biopsy (mm)	25.3±8.4	25.1±8.6	25.5±8.6	25.2±8.5	23.9±8.2	25.4±8.5
Number of portal tracts	21.0±8.3	20.7±8.7	21.1±8.7	20.9±8.4	20.3±8.6	20.6±8.4
Discordances between the 2 pathologists for fibrosis staging	N = 78 (29%)	N = 83 (31%)	N = 82 (32%)	N = 59 (31%)	N = 37 (31%)	N = 154 (30.5%)
Number (%) of discordances F1/F2	N = 46 (59%)	N = 47 (57%)	N = 45 (55%)	N = 29 (49%)	N = 20 (54%)	N = 72 (47%)
Consensual review of	30 F1 (65%)	30 F1 (64%)	29 F1 (64%)	20 F1 (69%)	13 F1 (65%)	48 F1 (67%)
	16 F2 (35%)	17 F2 (36%)	16 F2 (36%)	9 F2 (31%)	7 F2 (35%)	24 F2 (33%)

### Principal Component Analysis

ELFG was located differently in the PCA space with respect to Fibrotest®, Fibrometer® and Hepascore®, which were grouped close together and also not so close to Fibroscan™ (Figure S2).

### Medico-economic analysis

**Figure 3:**
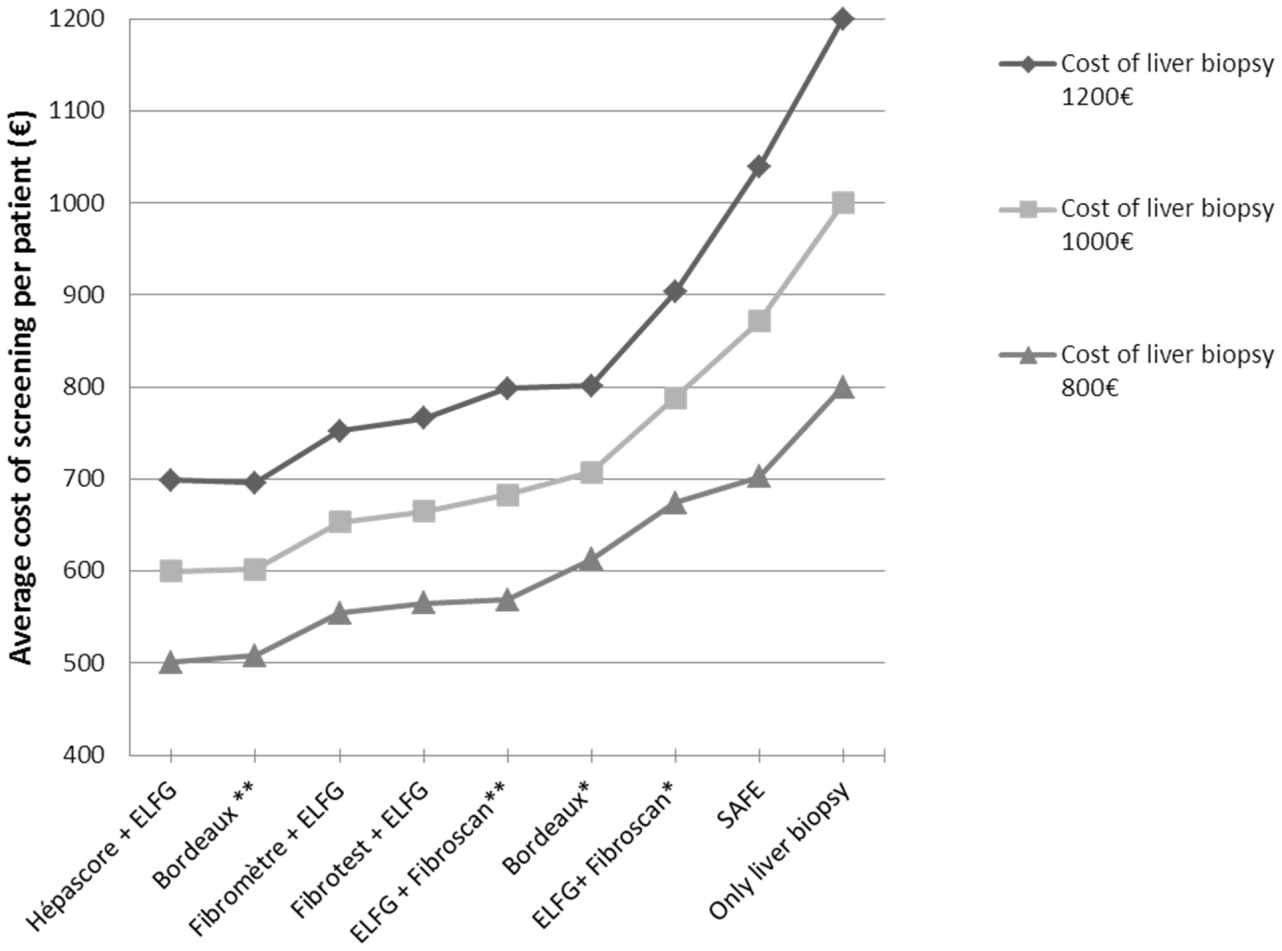
Economic analysis. Average cost of screening per patient (in euros) of the various combinations of tests, taking 3 levels of liver biopsy cost based on published data and the cost in our hospital: 800 Euros, 1,000 Euros and 1,200 Euros. *Cost of Fibroscan, for use equivalent to 10 acts per month. * *Cost of Fibroscan, for use equivalent to 32 acts per month.

When one test alone was inconclusive, the less expensive strategies were “Bordeaux” and Hepascore+ELFG. However the “Bordeaux” screening strategy includes Fibroscan^TM^ and the cost of Fibroscan^TM^ depends on the extent to which the instrument is used i.e. on the number of procedures per year (Figure 3). Most strategies that included the ELFG blood test were cheaper, except ELFG+Fibroscan^TM^ when the Fibroscan^TM^ device is infrequently used (less than 10 procedures per month).

## Discussion

Several algorithms have been proposed to improve the performance of the four validated tests (Fibrometer®, Fibrotest®, Hepascore® and Fibroscan^TM^) for the staging of significant fibrosis in CHC patients. The most used is Fibrotest®+Fibroscan^TM^. However, these algorithms have been constructed *a priori* without necessarily using all the tests available.

Here, we used an original methodology in which the algorithms were generated using an automated computerized induction method, which selected the appropriate tests and cut-offs from the full range of available tests. The cut-off for the first test was determined with a 90% NPV, and then the automated C4.5 induction program alone identified the best second test with a minimum of 80% PPV for the diagnosis of significant fibrosis (F≥2) without any intervention on the part of the investigators.

As shown in Figure 2, the better screening strategies for the diagnosis of significant fibrosis in “intention to diagnose” were Fibrometer®, Fibrotest® or Hepascore® in combination with the ELFG score. In “per-protocol analysis” the performance of the combination Fibroscan™+ELFG was similar to those combining two blood tests. The number of avoided liver biopsies varied between 50% and 55%. The diagnostic performance was better in terms of avoided liver biopsies compared to the usual combinations (Fibrometer®, Fibrotest® or Hepascore® plus Fibroscan^TM^). When we added a third test diagnostic performance was not improved, contrary to previously published results [22]. Our study clearly shows a better diagnostic performance than the SAFE algorithm [15] in terms of the number of avoided liver biopsies. In the “Bordeaux” algorithm [16] the NPV and PPV were lower than with the ELFG algorithm.

The cut-off for ELFG was the same whatever the first test used (−0.32). Several components of ELFG are direct markers of fibrosis and could explain the renewed interest in this test. Indeed, our “Principal Component Analysis” (Figure S2) describing the characteristics of different tests on two dimensions showed that ELFG provides different and complementary information to the other blood tests, closer to that of LSM.

In our main study the performance of Fibroscan™ was markedly reduced as results were unavailable or unreliable in more than 20% of cases, whereas the advantage of combining two blood tests for the diagnosis of significant fibrosis was highlighted.

In cases of discordant results, the choice of whether to perform a liver biopsy must be discussed because it is not a perfect “gold standard” [23], [24]. Any disagreements between the two pathologists in fibrosis staging were similar for all the combinations and occurred throughout the study population (Table 3). The quality of liver biopsy, the number of septa in the biopsy and the associated histological lesions could not explain these discrepancies. However the consensual review of the biopsy by both pathologists showed that the majority of patients (64–69%) had mild fibrosis (F1) when the discrepancy for staging was F1 or F2.

We also performed a cost-benefit analysis and compared the different strategies. To our knowledge, only one medico-economic study has been published in this field [20]. In that study the cost of liver biopsy was estimated 700 euros and the cost of Fibrotest® was 100 euros in absence of reimbursement by the social security. However the cost of Fibroscan^TM^ was not taken into account and the cost of the Bordeaux algorithm not analyzed. The SAFE strategy was cheaper than other algorithms. In the present study, that includes all blood tests and Fibroscan^TM^, we find the lowest cost strategies include ELFG. This result reinforces the interest of this test, as it is complementary to the others, as seen in the principal component analysis. From an economic perspective, the strategies that include Fibroscan^TM^ seem to be particularly interesting only when the rate of use of Fibroscan^TM^ is high. In other words, a hospital that doesn′t have a Fibroscan™ instrument should invest only if the frequency of use will be sufficient to offset the capital outlay.

In conclusion the use of the ELFG score following one of the three validated blood tests shows promise for improving the diagnosis of significant fibrosis in chronic hepatitis C and is cost-economic. Our algorithm using one of the validated blood tests (Fibrotest®, Fibrometer®, Hepascore®) is relatively cheap and ELFG could clearly replace Fibroscan™ allowing liver fibrosis to be staged in all CHC patients, including those who are overweight.

## Supporting Information

Figure S1 **Other previously published algorithms applied to Fibrostar database.**
(DOC)

Figure S2 **Principal Component Analysis of the five main tests.**
(TIF)

Table S1 Demographic, laboratory, and histological characteristics of the 507 CHC patients having all the blood tests and the 396 CHC patients with all the tests and reliable Fibroscan™.
(DOC)

Text S1 Details of laboratory tests with formulae for the calculation of the scores.
(DOC)

Text S2 Details of calculation of costs for the economic analysis.
(DOC)

Text S3 The ANRS HCEP-23 FIBROSTAR study group.
(DOC)
