# Supplementary figures and images for: Contribution of the ELFG Test in Algorithms of Non-Invasive Markers towards the Diagnosis of Significant Fibrosis in Chronic Hepatitis C
Source: PLoS One. 2013 Mar 21;8(3):e59088. doi: 10.1371/journal.pone.0059088 (PMC3605459; doi:10.1371/journal.pone.0059088)

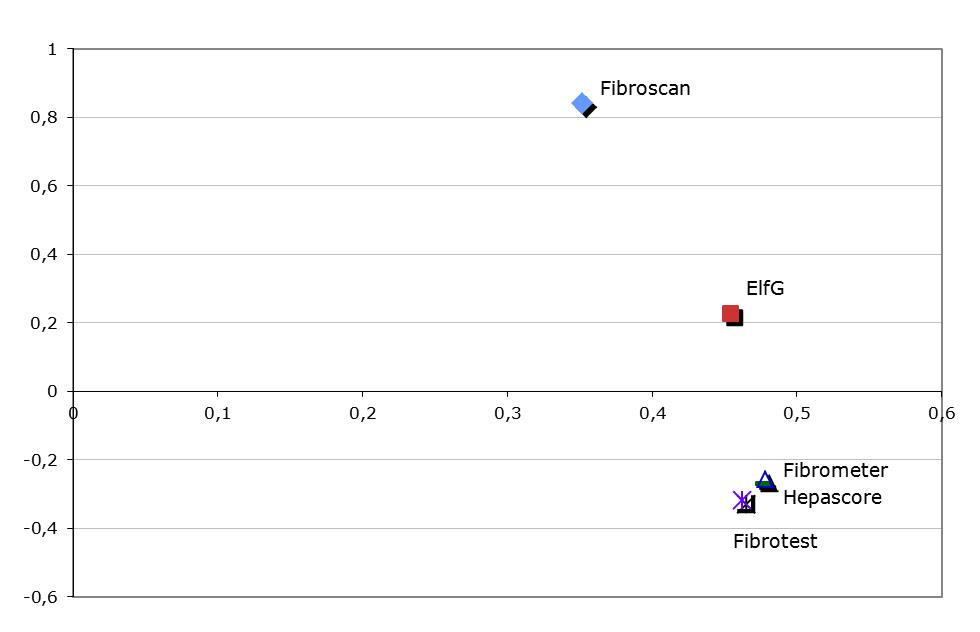

Supplement: Figure S2 — Principal Component Analysis of the five main tests. (TIF) [file pone.0059088.s002.tif]
