# Supplementary material for: Contribution of the ELFG Test in Algorithms of Non-Invasive Markers towards the Diagnosis of Significant Fibrosis in Chronic Hepatitis C
Source: PLoS One. 2013 Mar 21;8(3):e59088. doi: 10.1371/journal.pone.0059088 (PMC3605459; doi:10.1371/journal.pone.0059088)
Supplement: Table S1 — Demographic, laboratory, and histological characteristics of the 507 CHC patients having all the blood tests and the 396 CHC patients with all the tests and reliable Fibroscan™. (DOC) [file pone.0059088.s003.doc]

**Supporting Table S1.**

**Demographic, laboratory, and histological characteristics of the 507 CHC patients having all the blood tests** **and the 396 CHC patients with all the tests and reliable Fibroscan™**

| | **Characteristics** | | **n =507** | **n=396** |  | | --- | --- | --- | --- | --- | | Age (years) | | 51.6 ± 11.0 | 50.9 ± 10.7 |  | | Gender (N,%) | |  |  |  | | Male | | 307 (60.7%) | 239 (60.4%) |  | | Female | | 199 (39.3%) | 157 (39.6%) |  | | BMI (kg/m^2^) | | 25.0 ± 4.4 | 24.2 ± 3.4 |  | | Prothrombin Time (%) | | 94.4 ± 7.8 | 94.7 ± 7.9 |  | | Cholesterol (mmol/L) | | 1.8 ± 0.4 | 1.8 ± 0.4 |  | | Bilirubin (µmol/L) | | 12.3 ± 6.5 | 12.3 ± 6.7 |  | | ASAT (IU/L) | | 63.3 ± 43.5 | 62.5 ± 42.8 |  | | ALAT (IU/L) | | 88.3 ± 65.7 | 87.2 ± 65.0 |  | | GGT (IU/L) | | 96.7 ± 104.1 | 96.4 ± 98.8 |  | | Urea (mmol/L) | | 5.3 ± 2,6 | 5.3 ± 2.7 |  | | Platelet count (Giga/L) | | 215.2 ± 64.0 | 216.6 ± 65.2 |  | | Length of biopsy (mm) | | 25.4 ± 8.4 | 25.6 ± 8.4 |  | | Number of portal tracts | | 20.6 ± 8.4 | 20.9 ± 8.4 |  | | Liver fibrosis according to METAVIR fibrosis stage (%) | |  |  |  | | F0 | 34 (6.7%) | | 26 (6.6%) | | | F1 | 228 (45.0%) | | 188 (47.5%) | | | F2 | 91 (17.9%) | | 60 (15.1%) | | | F3 | 79 (15.6%) | | 65 (16.4%) | | | F4 | 75 (14.8%) | | 57 (14.4%) | | |  |
| --- | --- | --- | --- | --- | --- | --- | --- | --- | --- | --- | --- | --- | --- | --- | --- | --- | --- | --- | --- | --- | --- | --- | --- | --- | --- | --- | --- | --- | --- | --- | --- | --- | --- | --- | --- | --- | --- | --- | --- | --- | --- | --- | --- | --- | --- | --- | --- | --- | --- | --- | --- | --- | --- | --- | --- | --- | --- | --- | --- | --- | --- | --- | --- | --- | --- | --- | --- | --- | --- | --- | --- | --- | --- | --- | --- | --- | --- | --- | --- | --- | --- | --- | --- | --- | --- | --- | --- | --- | --- | --- | --- | --- | --- | --- | --- | --- | --- | --- | --- | --- | --- | --- | --- | --- | --- | --- | --- | --- | --- | --- | --- |

Results are expressed as mean ± one standard deviation

ASAT = aspartate aminotransferase; ALAT = alanine aminotransferase; GGT = gamma glutamyltranspeptidase
