# Supplementary material for: Contribution of the ELFG Test in Algorithms of Non-Invasive Markers towards the Diagnosis of Significant Fibrosis in Chronic Hepatitis C
Source: PLoS One. 2013 Mar 21;8(3):e59088. doi: 10.1371/journal.pone.0059088 (PMC3605459; doi:10.1371/journal.pone.0059088)
Supplement: Text S1 — Details of laboratory tests with formulae for the calculation of the scores. (DOC) [file pone.0059088.s004.doc]

**Supporting Information Text S1: Details of Laboratory tests**

**Abbreviations:**

ALT alanine aminotransferase; AST aspartate aminotransferase; GGT gamma glutamyl transpeptidase; PIIINP N-terminal peptide of type III procollagen; MM1 matrix metalloproteinase-1; TIMP1 tissue inhibitor of metalloproteinase-1

**Fibrotest® and its components**

The serum parameters of Fibrotest® were strictly measured according to the technical recommendations of the authors. using the same methods used to develop the test as described in their original publication [3]. Specific protein assays (α2-macroglobulin. haptoglobin. apolipoprotein A1) were measured by immunonephelemetric methods using a BN2 analyzer (Siemens Healthcare Diagnostics. Deerfield. USA). GGT. ALT and AST activities (IFCC methods at 37°C with pyridoxal phosphate for ALT and AST) and total bilirubin were measured using an Hitachi 917 analyzer (Roche Diagnostics. Mannheim. Germany) with reagents from the manufacturer and a CFAS (Calibrator for Automated Systems. Roche Diagnostics) calibration. Calculations of the scores were purchased from Biopredictive (Paris. France).

**Fibrometer® and its components**

The blood parameters were measured according to the technical recommendations. using methods previously used by the authors of this score [2]. Hyaluronan was assayed using HA Test Kit (Corgenix. Westminster. USA). As for Fibrotest**®.** α2-macroglobuline was measured using a BN2 analyzer and GGT. ALT. total bilirubin and urea were measured using an Hitachi 917 analyzer with reagents from the manufacturer and a CFAS calibration. The platelet count and prothrombin time were extemporaneously determined. The calculations of the scores were kindly provided by Prof. P. Calès (Angers. France).

**Forns score and its components**

GGT activity was measured as for Fibrotest**®** and Fibrometer**®**. Platelet count and cholesterol level were extemporaneously determined. The calculations were made according to the formula: FORNS score = 7.811 – 3.131 ln (platelet count (10^9^/L)) + 0.781 ln (GGT (IU/L)) + 3.467 ln (age (y)) – 0.014 (cholesterol (mg/dL)) as described [8].

**APRI and its components**

The AST activities were measured as for Fibrotest**®** and Fibrometer**®**. The platelet counts were extemporaneously determined. The scores were calculated according to the formula: APRI = 100 (AST/upper limit of normal (IU/L)) / Platelet count (10^9^/L). as described [10].

**MP3 and its components**

The N-terminal peptide of type III procollagen was assayed using an immunoradiometric assay (P3NP RIA. Orion Diagnostica. Espoo. Finland). metalloproteinase-1 using an ELISA assay (Human Pro-MMP-1 Quantikine Elisa Kit. R&D Systems. Abingdon. UK) and the scores were calculated as MP3 = 0.5903 Log PIIINP (ng/mL) – 0.1749 Log MM1 (ng/mL) as previously described [9].

**ELFG and its components**

Since the methods used by the authors are not available, the parameters included in this score were assayed using commercialized kits. The amino terminal peptide of type III procollagen was assayed using the P3NP RIA kit (Orion Diagnostica). The tissue inhibitor of metalloproteinase-1 was assayed using an Elisa kit (TIMP-1 Biotrak Elisa. GE healthcare Lifesciences. Orsay. France). The hyaluronic acid was assayed using the HA Test Kit (Corgenix). ELFG score was calculated as = - 0.014 ln (age (y)) +0.616 ln (hyaluronic acid (ng/mL)) + 0.586 ln (PIIINP (ng/mL)) + 0.472 ln (TIMP1 (ng/mL)) – 6.38 as described [11].

**Hepascore and its components**

Hyaluronic acid was assayed using the HA Test Kit (Corgenix). As for Fibrotest and Fibrometer. α2-macroglobuline was measured using a BN2 analyser (Siemens Diagnostic). GGT and total bilirubin were assayed using an Itachi 917 with CFAS calibration (Roche Diagnostic). The score was calculated as Hepascore = y/(1+y) with y = exp [-4.185818 – (0.0249 age (y)) + (0.7464 sex with M=1.F=0) + (1.0039 α2-macroglobuline (g/L)) + (0.0302 hyaluronic acid (µg/L)) + (0.0691 bilirubin (µmol/L)) – (0.0012 GGT (IU/L))] as described [1].

**FIB-4 and its components**

The AST and ALT activities were measured as for the other scores. The platelet counts were extemporaneously determined. The scores were calculated according to the formula: FIB4 = (age (y) x AST (IU/L)) / ((Platelet count (10^9^/L)) x (ALT (IU/L)^1/2^) as described [7].

**Collagen IV**

Serum collagen IV was measured using an immunoenzymatic assay (Serum Collagen IV EIA. Argutus Medical Ltd, Dublin, Ireland) [18].
