# Supplementary material for: Contribution of the ELFG Test in Algorithms of Non-Invasive Markers towards the Diagnosis of Significant Fibrosis in Chronic Hepatitis C
Source: PLoS One. 2013 Mar 21;8(3):e59088. doi: 10.1371/journal.pone.0059088 (PMC3605459; doi:10.1371/journal.pone.0059088)
Supplement: Text S2 — Details of calculation of costs for the economic analysis. (DOC) [file pone.0059088.s005.doc]

**Supporting Information N°2: Description of the calculation of costs of laboratory tests, of transient elastography (Fibroscan^TM^) and of liver biopsy**

**Cost of liver biopsy** To calculate the costs we separated the procedure of taking a biopsy sample from the histological analysis. For sampling we used the real hospital costs (hospitalization, staff costs and consumables) and for histological analysis we used the standard reimbursement rates of French Health Insurance (FHI).

The real cost of a liver biopsy for Grenoble hospital was 1148.5 €. In view of the variability in this cost from one establishment to another, described in the literature [e1,e2], we have estimated 3 levels of cost: 800 €, 1000€ e and 1200€.

**Table S2**: Cost of liver biopsy

|  | **Liver biopsy cost*  (€)** |
| --- | --- |
| Pre-biopsy check-up | 111 |
| Liver sampling | 1010.5 |
| Histological analysis | 27 |
| Total | 1148.5 |

* Valuation by hospital costs

***Cost of laboratory tests***
The cost of laboratory tests is the rate for reimbursement by the French health insurance and includes the cost of the analysis itself and the cost of using the algorithm for scoring.

**Table S3:** Cost of laboratory tests

|  | **Cost of blood screening test *  (€)** |
| --- | --- |
| Fibrotest® /Fibrometer ® | 91.6 |
| Hepascore | 41.6 |
| Apri | 14.4 |
| ELFG | 111 |

* Valuation by FHI costs

***Cost of transient elastography (FibroScan^TM^)***
Real cost at Grenoble University Hospital of measurement of liver stiffness by FibroScan^TM^:
The reconstruction of the actual cost of an act of transient elastography is based on the following data.

- Purchase price of FibroScan^TM^: € 79,500 including tax (purchase in 2005 by hospital).
- Maintenance: 2512 € including tax (Annual cost for hospital)
- Time and depreciation rates: respectively 7 years and 3%
- Logistics and hospital overheads: 20%
- Personnel Costs: 10 minute examination at 6 euros per exam. Examination performed by a radiologist, based on an annual salary of € 100,981 all inclusive (radiologist grade 7 on the grid of hospital clinicians). The hourly cost is 60 € / hour (1,670 hours/year)

**Table S4:** Cost of FibroScan^TM^ according to the number of procedures performed per month

| **FIBROSCAN^TM^ cost per procedure* (€)** | | | |
| --- | --- | --- | --- |
| Number of procedures per month | | | |
| **10** | **12** | **20** | **32** |
| 164.3 | 138.9 | 88.0 | 59.3 |

*** Valuation by hospital costs**

**References for supporting material N°2**

e1 Rolland-Burger L, Perrin JP, Vongmany N, *et al.* Marqueurs biologiques de la fibrose hépatique dans l'hépatite C. Rapport AP-HP. Paris: Comité d'évaluation et de diffusion des innovations technologiques (CEDIT); 2003. <http://ancien-cedit.aphp.fr/servlet/siteCedit?Destination=reco&numArticle=02.08/Re1/04>

e2 Service évaluation des actes professionnels, Service des recommandations professionnelles, Service médico-économique et santé publique. Méthodes d’évaluation de la fibrose hépatique au cours des hépatopathies chroniques, rapport HAS, Décembre 2006. <http://www.has-sante.fr/portail/upload/docs/application/pdf/rapport_fibrose.pdf>
