# Supplementary material for: Contribution of the ELFG Test in Algorithms of Non-Invasive Markers towards the Diagnosis of Significant Fibrosis in Chronic Hepatitis C
Source: PLoS One. 2013 Mar 21;8(3):e59088. doi: 10.1371/journal.pone.0059088 (PMC3605459; doi:10.1371/journal.pone.0059088)
Supplement: Text S3 — The ANRS HCEP-23 FIBROSTAR study group. (DOC) [file pone.0059088.s006.doc]

**Supplementary material Text S3**

T**he ANRS HCEP-23 FIBROSTAR study group**

**Promotor:**

L. Allain, French Agency for Research on AIDS and Viral Hepatitis (A.N.R.S),

**Hepatologists:**

J-P. Zarski, V. Leroy, Centre Hospitalier Universitaire de Grenoble.

R. Poupon, A. Poujol-Robert, Hôpital Saint-Antoine, Assistance Publique-Hôpitaux de Paris.

A. Abergel, Centre Hospitalier Universitaire de Clermont-Ferrand.

J.P. Bronowicki, Hôpital de Brabois, Centre Hospitalier Universitaire de Nancy.

J.P. Vinel, S. Metivier, Hôpital Purpan, Centre Hospitalier Universitaire de Toulouse.

V. De Ledinghen, Hôpital Haut Levêque, Centre Hospitalier Universitaire de Bordeaux.

O. Goria, Centre Hospitalier Universitaire de Rouen

M. Maynard-Muet, C. Trepo, F. Bailly, Hôtel Dieu, Hospices Civils de Lyon.

Ph. Mathurin, Centre Hospitalier Universitaire de Lille

D. Guyader, H. Danielou, Hôpital Pontchaillou, Centre Hospitalier Universitaire de Rennes.

O. Rogeaux, Centre Hospitalier de Chambéry.

S. Pol, Ph. Sogni, Hôpital Cochin, Assistance Publique-Hôpitaux de Paris.

A. Tran, Hôpital De l’Archet, Centre Hospitalier Universitaire de Nice

P. Calès, F Oberti, F Lunel-Fabiani, Centre Hospitalier Universitaire d’Angers

P. Marcellin, T. Asselah, Hôpital Beaujon, Clichy, Assistance Publique-Hôpitaux de Paris.

M. Bourliere, V. Oulès, Hôpital Saint Joseph, Assistance Publique-Hôpitaux de Marseille

D. Larrey, Centre Hospitalier Universitaire de Montpellier

F. Habersetzer, Centre Hospitalier Universitaire de Strasbourg

M. Beaugrand, Hôpital Jean Verdier, Assistance Publique-Hôpitaux de Paris.

**Biologists:**

R-C. Boisson, Centre Hospitalier Lyon Sud, Hospices Civils de Lyon.

M-C. Gelineau, B. Poggi, Hôtel Dieu, Hospices Civils de Lyon.

J-C. Renversez, Candice Trocmé, Centre Hospitalier Universitaire de Grenoble.

J. Guéchot, E. Lasnier, M. Vaubourdolle, Hôpital Saint-Antoine, Assistance Publique-Hôpitaux de Paris

H. Voitot, Hôpital Beaujon, Assistance Publique-Hôpitaux de Paris.

A. Vassault, Hôpital Necker, Assistance Publique-Hôpitaux de Paris.

A. Rosenthal-Allieri, Centre Hospitalier Universitaire de Nice.

A. Lavoinne, F. Ziegler, Centre Hospitalier Universitaire de Rouen.

M. Bartoli, C. Lebrun, Centre Hospitalier de Chambéry.

A. Myara, Groupe Hospitalier Paris Saint-Joseph, Paris.

F. Guerber, A. Pottier, Laboratoire Elibio -Groupe Oriade, Vizille, La Mure.

**Pathologists:**

E-S. Zafrani, Hôpital Henri Mondor, Créteil, Assistance Publique-Hôpitaux de Paris.

N. Sturm, Centre Hospitalier Universitaire de Grenoble.

**Methodologists and administrators:**

A. Bechet, J-L Bosson, A. Paris, S. Royannais, Centre d’Investigation Clinique, Centre Hospitalier Universitaire de Grenoble.

A. Plages, HépatoGastroEntérologie, Centre Hospitalier Universitaire de Grenoble

S. Zafrani, Hôpital Henri Mondor, AP-HP, Créteil.
