# Supplementary material for: Contribution of the ELFG Test in Algorithms of Non-Invasive Markers towards the Diagnosis of Significant Fibrosis in Chronic Hepatitis C
Source: PLoS One. 2013 Mar 21;8(3):e59088. doi: 10.1371/journal.pone.0059088 (PMC3605459; doi:10.1371/journal.pone.0059088)
Supplement: Supplementary file 6 [file pone.0059088.s001.doc]

“Bordeaux” algorithm

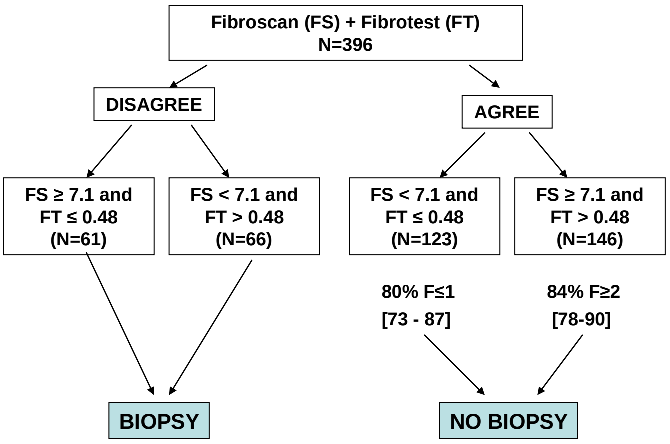

Avoided biopsies 123 + 146 = 269   (68% [63-73])

“SAFE” algorithm

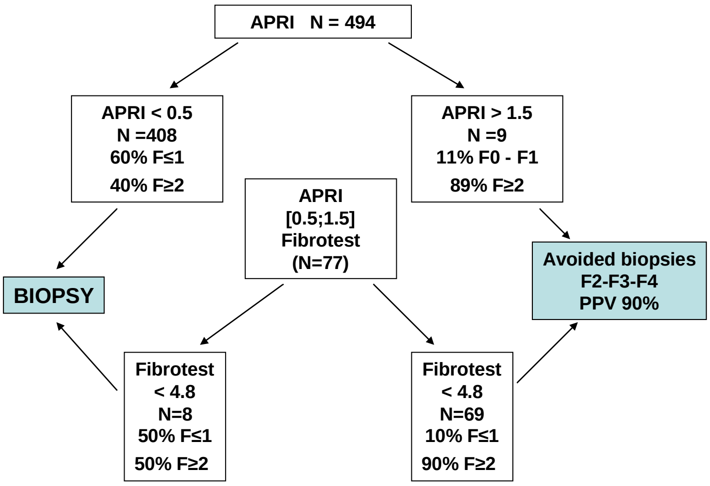

Avoided biopsies 69 + 9 = 78   (16% [13-19])
